# Health-related quality of life among children with mental health problems: a population-based approach

**DOI:** 10.1186/1477-7525-10-73

**Published:** 2012-06-18

**Authors:** Michelle Dey, Meichun Mohler-Kuo, Markus A Landolt

**Affiliations:** 1 Institute of Social and Preventive Medicine, University of Zurich, Hirschengraben 84, 8001, Zurich, Switzerland; 2 Department of Psychosomatics and Psychiatry, University Children’s Hospital Zurich, Steinwiesstrasse 75, 8032, Zurich, Switzerland; 3 Children’s Research Center, University Children’s Hospital Zurich, Steinwiesstrasse 75, 8032, Zurich, Switzerland

**Keywords:** Mental disorders, Attention deficit disorder, Quality of life

## Abstract

**Background:**

Children with mental health problems have been neglected in health-related quality of life (HRQOL) studies*.* Therefore, the aims of the current study were 1) to assess the influence of the presence of mental or physical health problems on HRQOL; and 2) to analyze the effects of item overlap between mental health problems and HRQOL-measurements.

**Methods:**

Proxy- and self-rated HRQOL (KIDSCREEN-27) of children 9–14 years old was assessed across children with mental health problems (*n* = 535), children with physical health problems (*n* = 327), and healthy controls (*n* = 744). Multiple linear regression analyses were conducted with health status, severity of symptoms, status of medication use, gender and nationality as independent, and HRQOL scores as dependent variables. The effects of item overlap were analyzed by repeating regression analyses while excluding those HRQOL items that contextually overlapped the most frequently-occurring mental health problem (attention deficits).

**Results:**

Severity of symptoms was the strongest predictor of reduced HRQOL. However, all other predictors (except for the status of medication use) also contributed to the prediction of some HRQOL scores. Controlling for item overlap did not meaningfully alter the results.

**Conclusions:**

When children with different health constraints are compared, the severity of their particular health problems should be considered. Furthermore, item overlap seems not to be a major problem when the HRQOL of children with mental health problems is studied. Hence, HRQOL assessments are useful to gather information that goes beyond the clinical symptoms of a health problem. This information can, for instance, be used to improve clinical practice.

## Background

‘Health-related quality of life’ (HRQOL) can be described as a subjective, multidimensional and dynamic construct that comprises physical, psychological and social functioning and that is, among other things, influenced by the health condition of the particular person [1]. To date, more studies have been conducted evaluating the HRQOL of individuals with *physical* than with *mental health conditions*[2]. Additionally, more HRQOL studies have targeted *adults* than *children*[3].

In a recent review article [4], children with *various mental health conditions* were found to exhibit compromised HRQOL relative to *healthy peers*. The largest effect sizes (ES) have been identified for the total HRQOL score and various psychosocial scales, whereas the ES for physical scales generally have been smaller. Parent ratings of a child’s HROQL often were most affected in the psychosocial subscale most closely related to the particular mental health condition. One explanation for this observation is so-called *item overlap*, which is defined as content similarities between HRQOL items and the conceptualization of a particular mental health condition. When such overlap exists, HRQOL and mental health problems are inevitably related [5]. To date, most studies have failed to control for item overlap [4]. However, Sawyer et al. [6] analyzed this issue and observed similar relationships between mental disorders and HRQOL, even after controlling for item overlap. Nevertheless, the effects of this mechanism should be evaluated further [2,5].

Besides non-consideration of item overlap, the following limitations of previous research were extracted in the above-mentioned review article [4]: first, many authors only used proxy ratings, even though the subjectivity of HRQOL actually demands self-rating [7] whenever the child’s cognitive abilities and particular mental health condition permit [5,8]. Second, many studies failed to capture whether or not the child was receiving medication for the specific mental health condition, even though it is possible that such treatment affects HRQOL. Furthermore, other potentially-influential variables (e.g., severity of the health condition) were often not included in statistical analyses. Third, existing studies are limited by primarily relying on clinical samples. The utilization of such samples may lead to biased results, because mentally ill children who concurrently have compromised HRQOL may be more frequently referred to or treated in clinics than children with mental health problems lacking HRQOL impairment [6,9]. Studies that use population-based samples should be used to verify any results obtained from clinical samples [4]. The few existing studies that have utilized population-based approaches have identified reduced HRQOL (especially within psychosocial (sub)scales) among children with mental health problems [6,10]. Finally, only a limited number of studies have compared children with mental and physical health conditions with regard to their HRQOL (e.g., [6,10-14]). Even though the results of these studies are not completely consistent, Danckaerts et al. [8] summarized various studies and concluded that the overall HRQOL score is reduced to the same extent in children with mental and physical health conditions, whereas psychosocial HRQOL domains are more compromised in children with mental, and physical HRQOL domains in children with physical health problems.

Addressing existing research gaps and the aforementioned limitations, the aims of the present study were two-fold: First, we aimed to assess the influence of mental health problems on proxy- and self-rated HRQOL scores in a population-based sample. For comparison’s sake, we also intended to assess the influence of physical health problems on HRQOL. Hereby, other potentially-influencing variables (severity of symptoms, status of medication use) were taken into account. Second, we aimed to examine whether item overlap effects the association between mental health problems and HRQOL.

## Methods

### Study procedure

The present study used data from the National Survey of Children with Special Health Care Needs in Switzerland which included a sample of children ages 9–14 from all 26 cantons in Switzerland. We chose children under 15 years, as most health surveys have targeted respondents 15 years old or older. Furthermore, selecting this age group, as opposed to much younger children, allowed us to obtain a HRQOL assessment from both the parents and the children themselves. The protocol was approved by the ethics committee of the Canton of Zurich. A two-stage, population-based sampling method was used to obtain a representative sample (for further details see [15]). The original sample consisted of 16,496 children and their parents.

The main purpose of phase I was to screen the children for *special health care needs* (see measurements). We received screening information about 10,830 children (response rate = 65.7%). Based upon the screening, 1,492 children were classified as *children with special health care needs* (*CSHCN*), 9,294 as children without special health care needs (*healthy controls*) and 44 children were not classifiable due to *missing* data. The latter were excluded from further analyses. Based upon additional information that was gathered during this phase, the CSHCN were subdivided into *CSHCN with mental health problems* (*n* = 919), *CSHCN with physical health problems* (*n* = 543) and *CSHCN with no classifiable main health problem* (*n* = 30). The latter were excluded from further analyses.

The main goal of phase II was to collect information about the self- and proxy-rated HRQOL of *all CSHCN* and a group of *randomly selected healthy controls*. However, not all CSHCN could be re-contacted (if the parents refused to be re-contacted after screening or if the survey material for phase I was returned after the data collection component of phase II was already completed). Altogether, 2,658 HRQOL questionnaires were sent out immediately after screening (881 to CSHCN with mental health problems, 524 to CSHCN with physical health problems, and 1,253 to healthy controls), with seven parent–child pairs excluded because they were no longer contactable (2 CSHCN with mental health problems, 1 CSHCN with a physical health problem, and 4 healthy controls). We received questionnaires back from 60.6% of the parents and/or children (see next section) of the remaining 2,651 parent–child pairs.

In phases I and II, it was emphasized that participation was voluntary. By answering the questions, the parents and/or children provided informed consent.

### Sample

We examined children living in Switzerland between the ages of 9 and 14 years. The present study only included children from phase II for which information about HRQOL was provided (self- and/or parent-rating). Altogether, 535 *CSHCN with mental health problems,* 327 *CSHCN with physical health problems* and 744 *healthy controls* were included in the current analysis. The demographic characteristics of the three health status groups are presented in Table 1. The most frequently mentioned mental health problems of CSHCN were attention deficits (*n* = 204), learning difficulties (*n* = 131) and conduct problems (*n* = 53). CSHCN with physical health problems most frequently had diseases of the respiratory system (*n* = 106; e.g. asthma), diseases of the musculoskeletal system and connective tissue (*n* = 47; e.g. scoliosis) and diseases of the nervous system (*n* = 31; e.g. epilepsy).

**Table 1:** Demographic characteristics of the three health status groups and health characteristics of children with special health care needs (CSHCN)

	**CSHCN with mental health problems**	**CSHCN with physical health problems**	**Healthy controls**	***X*^2^**	**df**	***p***
	**(*n* = 535)**	**(*n* = 327)**	**(*n* = 744)**			
Age, years (mean ± SD)	11.39 ± 1.45	11.51 ± 1.54	11.46 ± 1.53	8.281	10	.601
Gender (% male)	65.4	56.0	47.0	42.624	2	*p* < 0.001
Nationality (% Swiss)	93.6	94.8	89.5	11.644	2	0.003
Severity of the main health problem of CSHCN						
Very low severity (%)	4.7	13.5		47.401	4	*p* < 0.001
Low severity (%)	17.6	29.1				
Average severity (%)	48.6	39.8				
High severity (%)	23.6	12.8				
Very high severity (%)	5.6	4.9				
Medication for the main health problem (% yes)	37.6	65.1		61.791	1	*p* < 0.001

As demonstrated in Table 1, the three health status groups differed in terms of their distribution by gender and nationality. CSHCN with mental or physical health problems further differed with respect to the severity of the main health problem and status of medication use. Thereby, the *(very) low severity* groups had a disproportionate large number of CSHCN with physical health problems relative to CSHCN with mental health problems, whereas the opposite pattern was identified among the *average* to *very high severity* groups. CSHCN with physical health problems were more frequently on medication than CSHCN with mental health problems.

### Measures

*Special health care needs*: To assess special health care needs, the parent-reported *CSHCN Screener*[16] was used. According to this well-validated and widely-used instrument, a child is classified as *having special health care needs* if he/she presently experiences at least one of five health consequences (e.g., item 1: the need for or use of prescribed medicine; item 5: the need for or use of treatment or counseling for emotional, developmental or behavioral problems) that is due to a health condition which has lasted or is expected to last at least 12 months. If the child did not experience any health consequences, he/she was classified as a *healthy control*.

*Classification of CSHCN:* After screening for CSHCN, the parents were asked to describe the *main health problem* (open answer format) associated with those special health care needs. The responses were coded according to the *International Classification of Disease and Related Health Problems* (ICD-10 [17]) from which the following two subject groups were created: 1) *CSHCN with mental health problems*, if the main health problem associated with having special health care needs could be assigned to one of the disorders listed in Chapter V (‘Mental and behavioral disorders’) of the ICD-10; and 2) *CSHCN with physical health problems,* if the main health problem associated with special health care needs could be assigned to Chapter I to IV or VI to XIX of the ICD-10. Altogether, 68 CSHCN could not be assigned to either CSHCN with a mental health problem or physical health problem (e.g., because the parents did not report a specific health problem). These children were assigned to CSHCN with a mental health problem if item 5 of the CSHCN Screener was positive (the need for or use of treatment or counseling for emotional, developmental or behavioral problems) [18]. Accordingly, an additional 38 children were assigned to the group of C*SHCN with a mental health problem*. The remaining 30 were excluded from further analysis.

*Status of medication use:* According to responses to the first item of the CSHCN Screener, the ‘status of medication use’ was dichotomized as yes/no.

*Severity of the main health problem:* The parents of CSHCN were asked to rate the severity of their child’s main health problem on a five-point scale, ranging from ‘1’ (not at all severe) to ‘5’ (very severe).

*HRQOL*: The proxy- and self-reported *KIDSCREEN-27*[19] was used to assess HRQOL. This instrument is applicable to children ages 8 to 18 years and has been validated internationally. It contains 5 subscales, namely ‘physical well-being’, ‘psychological well-being’, ‘autonomy & parent relation’, ‘social support & peers’ and ‘school environment’. A 5-point response scale is used for rating, with scores ranging from ‘1’ (not at all/never) to ‘5’ (extremely/always). Because the five subscales differ in the number of items, the sum scores for each subscale were standardized to a scale ranging from 0 to 100, whereby higher scores indicate better HRQOL. Furthermore, in accordance with the manual, a total HRQOL score was calculated that was based on the sum of 10 items. This summation score was also standardized to a scale ranging from 0 to 100. For the overall sample, internal consistency (Cronbach’s α [20]) ranged from 0.78 to 0.88 for parental ratings and from 0.74 to 0.83 for child ratings.

### Statistical analyses

All analyses were conducted using SPSS Version 17.0 for Macintosh [21]. Analyses were performed with two-sided tests and *p* < .05 was considered significant.

Differences in the demographic and health characteristics among the three health status groups (CSHCN with mental health problems, CSHCN with physical health problems, and healthy controls) were compared using *χ*^2^ analyses.

Multiple linear regression analyses were used to assess the association between HRQOL (proxy- and self-rated HRQOL scores) and related predictors, which included health status, severity of the main health problem, and status of medication use. Variance inflation factors (VIF) were used to assess whether multicollinearity between the independent variables existed. A VIF ≥ 10 was interpreted as indicating the presence of multicollinearity [22].

To analyze the effects of *item overlap*, we selected the largest subgroups of CSHCN with mental health problems: children with attention deficits (*n* = 204). We therefore repeated the above-mentioned multiple linear regression analyses for this subgroup 1) without controlling for item overlap; 2) excluding the HRQOL item exhibiting the greatest degree of item overlap with attention deficits (‘Have you/has your child been able to pay attention?’); and 3) excluding the two HRQOL items most closely related to attention deficits (additionally excluding the item ‘Have you/has your child got on well at school?’). Because ‘school environment’ was the only subscale that was affected by controlling for item overlap, only the scores of this subscale are reported.

## Results

### Descriptive HRQOL data by health status group

The means and SD of proxy- and self-rated HRQOL by health status group are presented in Table 2. The means were always highest for healthy controls. For the ‘physical well-being’ subscale, CSHCN with physical health problems had the lowest scores, whereas CSHCN with mental health problems had the lowest scores for all other HRQOL (sub)scales.

**Table 2:** Means and standard deviations for self- and parent-reported KIDSCREEN-27 scores

	**CSHCN with mental health problems**	**CSHCN with physical health problems**	**Healthy controls**
	**mean (SD)**	**mean (SD)**	**mean (SD)**
*Parent report*			
Physical well-being	72.19 (16.83)	69.16 (17.40)	78.39 (14.18)
Psychological well-being	73.90 (14.13)	77.75 (13.05)	82.09 (10.35)
Autonomy & parent relation	73.32 (13.67)	75.82 (12.92)	77.93 (12.86)
Social support & peers	64.01 (21.24)	66.01 (20.16)	72.17 (17.03)
School environment	65.49 (18.24)	76.34 (14.95)	78.60 (14.37)
Total HRQOL score	70.43 (12.36)	74.94 (11.21)	79.14 (10.07)
*Child report*			
Physical well-being	73.25 (17.00)	71.77 (16.30)	79.41 (14.24)
Psychological well-being	80.45 (14.79)	82.97 (13.34)	85.97 (11.43)
Autonomy & parent relation	77.59 (15.88)	82.06 (13.60)	83.11 (14.03)
Social support & peers	75.35 (22.28)	77.88 (19.75)	82.34 (16.50)
School environment	72.57 (18.64)	79.39 (14.74)	80.63 (15.79)
Total HRQOL score	76.41 (13.37)	80.53 (11.56)	83.13 (11.04)

*Note*: The number of subjects (*n*) varies between the subscale and total HRQOL scores due to missing data. The largest *n* for parent ratings was 520 for CSHCN with mental health problems, 321 for CSHCN with physical health problems, and 732 for healthy controls. The largest *n* for child ratings was 454 for CSHCN with mental health problems, 278 for CSHCN with physical health problems and 686 for healthy controls.

### Multiple linear regression analyses

Besides health status, the severity of the main health problem, and the status of medication use, we also included gender and nationality as independent variables for multiple linear regression analyses, because these variables differed by health status group (see Table 1). Multiple linear regression results are presented in Table 3. No indicators of multicollinearity were present (all VIF factors ≤ 10).

**Table 3:** Multiple linear regression analyses on parent- and child-reported HRQOL (total HRQOL and subscales)

	**Physical well-being**		**Psychological well-being**		**Autonomy & parent relation**		**Social support & peers**		**School environment**		**Total HRQOL**	
	**Parent report**	**Child report**	**Parent report**	**Child report**	**Parent report**	**Child report**	**Parent report**	**Child report**	**Parent report**	**Child report**	**Parent report**	**Child report**
	**β**	**β**	**β**	**β**	**β**	**β**	**β**	**β**	**β**	**β**	**β**	**β**
Health status												
Healthy controls (reference)												
CSHCN with mental health problems	.222^***^	.047	.108	.032	.016	-.096	.042	.026	-.106	-.167^**^	.042	-.113
CSHCN with physical health problems	.069	-.026	.179^***^	.084	.063	.022	.042	.032	.138^**^	-.004	.150^**^	.018
Severity	-.477^***^	-.283^***^	-.456^***^	-.257^***^	-.207^***^	-.091	-.268^***^	-.227^***^	-.259^***^	-.069	-.432^***^	-.172^**^
Medication	.005	.011	-.033	-.028	.015	.009	.018	.041	-.021	.040	-.017	.008
Female gender	-.116^***^	-.102^***^	-.050^*^	-.075^**^	.010	.033	.040	.052^*^	.102^***^	.041	-.006	-.039
Non-Swiss nationality	-.057^*^	-.056^*^	-.028	-.024	-.037	-.086^***^	-.008	-.031	.009	.030	-.015	-.031
***R***^***2***^***adjusted***	.111	.069	.119	.048	.029	.033	.048	.035	.141	.046	.138	.061
***F***	32.86	18.10	36.28	12.89	8.57	8.91	14.05	9.50	43.95	12.15	41.78	15.87
**df**	6, 1528	6, 1387	6, 1557	6, 1409	6, 1533	6, 1366	6, 1547	6, 1398	6, 1566	6, 1389	6, 1527	6, 1364
***p***	<.001	<.001	<.001	<.001	<.001	<.001	<.001	<.001	<.001	<.001	<.001	<.001

*Note*: Both significant and non significant standardized betas are reported; * = *p* ≤ .05; ** = *p* ≤ .01 *** = *p* ≤ .001; Coding severity: no problem (healthy controls; 0) – very severe (5).

For all HRQOL scores (except for self-rated ‘autonomy & parent relation’ and ‘school environment’), the most important predictor of reduced HRQOL was the severity of the main health problem. In contrast, use of medication was not associated with any HRQOL scores. All other independent variables were significantly associated with some HRQOL scores. Within the multiple regression models, the presence of mental health problems predicted better parent-reported ‘physical well-being’, and poorer self-reported ‘school environment’. The presence of a physical health problem was significantly associated with better parent-reported ‘psychological well-being’, ‘school environment’ and ‘total HRQOL’. Female gender predicted reduced proxy- and self-reported ‘physical well-being’ and ‘psychological well-being’ and increased scores for self-reported ‘social support & peers’ and parent-reported ‘school environment’. Non-Swiss nationality predicted reduced scores for proxy- and self-rated ‘physical well-being’ and self-reported ‘autonomy & parent relation’ subscales.

All regression models were significant at *p* < .001 and accounted for 2.9% to 13.8% of the variance in the parent-reported HRQOL scores, and for 3.3% to 6.9% of the variance in the self-reported HRQOL scores.

### Item overlap

All three sets of analyses (without correction for item overlap; excluding one overlapping item; and excluding two overlapping items) are presented in Table 4. No indicators of multicollinearity were identified (all VIF factors ≤ 10). The influence of the variable ‘children with attention deficits’ on ‘school environment’ decreased with increasing control for item overlap, as indicated by decreasing standardized betas. However, these changes were only small in magnitude.

**Table 4:** Multiple linear regression analyses on parent- and child-reported ‘school environment’ with and without controlling for item overlap

	**Without correction for item overlap**		**Excluding one overlapping item**		**Excluding two overlapping item**	
	**Parent report**	**Child report**	**Parent report**	**Child report**	**Parent report**	**Child report**
	**β**	**β**	**β**	**β**	**β**	**β**
Health status						
Healthy controls (reference)						
Children with attention deficits	-.190^**^	-.262^***^	-.181^**^	-.221^**^	-.134^*^	-.158^*^
CSHCN with physical health problems	.085	-.108	.054	-.082	.055	-.072
Severity	-.222^***^	.036	-.172^*^	.012	-.142^*^	.017
Medication	.047	.087^*^	.047	.076	.010	.060
Female gender	.093^***^	.042	.090^***^	.054	.105^***^	.063^*^
Non-Swiss nationality	.014	.054	.016	.053	.034	.065^*^
***R***^***2***^***adjusted***	.128	.040	.095	.035	.072	.020
***F***	31.51	8.82	23.00	7.76	17.26	4.78
**df**	6, 1246	6, 1115	6, 1246	6, 1115	6, 1246	6, 1115
***p***	<.001	<.001	<.001	<.001	<.001	<.001

*Note:* Both significant and non significant standardized betas are reported; * = *p* ≤ .05; ** = *p* ≤ .01 *** = *p* ≤ .001; Coding severity: no problem (healthy controls; 0) – very severe (5).

## Discussion

The purpose of this article was to evaluate the HRQOL of children with mental health problems in-depth. Hereby, some of the research gaps and limitations of existing studies were considered: most important was that 1) a large population-based sample was used; 2) the issue of item overlap was addressed; and 3) in addition to the CSHCN with mental health problems and healthy controls, children with physical health problems were included.

The descriptive statistics of our study are partially consistent with the pattern that was proposed in a review article [8]: That is, the lowest means for *psychosocial HRQOL domains* were identified among children with mental health problems, whereas the lowest means for *physical HRQOL domains* were apparent among children with physical health problems. However, when one only considers the descriptive statistics, it seems that our results contradict the proposition that *overall HRQOL* is equally compromised in children with mental and physical health problems [8]. This being said, the finding of lower total HRQOL scores among CSHCN with mental versus physical health problems was probably due to the severity of the main health problem being greater for the former group. Accordingly, multiple regression analyses indicated that reduced HRQOL was primarily associated with increased severity of the main health problem. This effect was also apparent for total HRQOL.

Despite the clear importance of the severity of the main health problems in predicting HRQOL, mental and physical health problems also contributed to the prediction of some HRQOL scores. For instance, the presence of mental health constraints was significantly associated with a poorer ‘school environment’ when self reports were considered, and tended towards poorer ‘school environment’ when parent reports were used. The effect on this subscale may be due to the composition of our sample, as the most frequently-reported mental health problems were attention deficits, learning difficulties and conduct problems, thus problems that share their strong impact upon school context. It seems that this impact was not entirely attributable to content similarities between the conceptualization of mental health problems and HRQOL items, because our results remained largely unchanged even when we controlled for item overlap (comparable to findings by Sawyer et al. [6]). Other psychosocial subscales (e.g., ‘psychological well-being’) were not reduced in CSHCN with mental health problems in multiple regression models. This might be because this subscale was less directly affected by the most frequently-represented mental health problems in our sample.

That the presence of a mental health problem was associated with enhanced parent-reported ‘physical well-being’, whereas the presence of a physical health problem predicted higher parent-reported ‘psychological well-being’ and ‘school environment’ may be due to compensatory and overly-positive ratings by parents in those HRQOL domains that are not directly related to the particular health constraint [23].

The status of medication use had no influence on HRQOL. This might be due to our inclusion of a variety of different health constraints in our study that were not treated uniformly. Hence, it is possible that the positive effects of some drugs on HRQOL were overlaid by the negative effects of others (e.g., drugs that have severe side effects).

The two included demographic characteristics also exerted an influence on some HRQOL scores in the multiple linear regression models. First and comparable with other studies (e.g., [24]), female gender was associated with reduced ‘physical well-being’ and ‘psychological well-being’, as well as with increased ‘social support & peers’ and ‘school environment’. Second, non-Swiss nationality had a significant negative impact upon self- and parent-reported ‘physical well-being’ as well as on self-reported ‘autonomy & parent relation’. This result (especially the former) can be explained by the compromised health status of the non-Swiss population (see, for instance [25]) that leads to reduced HRQOL. However, it also may be due to a more negative assessment of the same health status by non-Swiss relative to Swiss subjects.

Despite the strengths of our study, some limitations must be considered. Similar to other population-based studies (e.g., [10]), the most important limitation was that precise diagnostic information about the main health problem was missing (i.e., CSHCN were classified primarily based upon the parent-reported *main health problem*). However, population-based studies generally uncover similar results as those in which children are diagnosed through a specialist [10]. Furthermore the utilization of clinical samples may lead to biased results [6,9]. Hence, population-based studies seem to be a worthwhile supplementation to studies with clinical samples [4]. It can be argued that the CSHCN who were not classifiable based upon their main health problem were classified according to answers to the *fifth item of the CSHCN Screener*. We cannot rule out that the *need for treatment and/or counseling for emotional, developmental or behavioral problems* was the consequence of a significant physical health problem. However, additional analyses indicated that CSHCN with mental health problems who were classified according to the parent-reported main health problem were similar, in terms of their HROQL, as CSHCN with mental health problems classified according to the fifth item of the CSHCN Screener. Hence, we assume that applying this second method was valid.

A second study limitation was that we had no information about co-morbid conditions, even though it is possible that these conditions might contribute to the prediction of HROQL.

Third, most of the independent variables (e.g. the health status) were based upon answers provided by the parents. Hence, that the explained variances in multiple linear regression models were larger when HRQOL was rated by parents than by children might be explained through shared-rater method variance.

A fourth limitation is that we used standardized HRQOL scores instead of the Rasch-scaled HRQOL scores proposed by the KIDSCREEN-27 developers. We decided against using Rasch-scaled HRQOL scores because they are difficult to interpret. To deal with this problem, the developers proposed recoding these values into T-scores, a transformation that is based upon norm values. However, only the German-speaking part of Switzerland was included to obtain these norm data, whereas we also drew subjects from the French- and Italian-speaking regions of Switzerland.

## Conclusions

The present paper demonstrates the significant contribution of the *severity* of a child’s main health problem to predicting HRQOL, a contribution that has implications for the interpretation of the results of other studies. That is, HRQOL differences between children with mental and physical health problems could be more or less pronounced when the severity of health problems is taken into account. Besides the severity of the main health problem, additional variables were important in predicting HRQOL. One important finding was that the presence of a mental health problem predicted a poorer ‘school environment’, a HROQL domain that was most closely related to the most-frequently represented mental health problems (attention deficits, learning difficulties, and conduct problems). This finding seems not to be solely attributable to item overlap between mental health problems and HRQOL items, because our results remained much the same when we controlled for item overlap. Hence, HRQOL assessments are useful when attempting to gather wide-ranging information about CSHCN. This information, which goes beyond the clinical symptoms of mental health problems, can be used in many ways: 1) to expand knowledge about the impact of particular mental health constraints; 2) to improve clinical practices (e.g., by considering compromised HRQOL domains in therapy); and 3) to adapt public health practices (e.g., by considering a broad range of different health conditions and comparing their HRQOL constraints, so as to adequately plan services) [2].

## Abbreviations

ADHD, Attention-deficit/hyperactivity disorder; CSHCN, Children with special health care needs; HRQOL, Health-related quality of life; ICD-10, International Classification of Disease and Related Health Problems; VIF, Variance inflation factors; EF, Effect sizes; ES, Effect sizes.

## Competing interests

The authors declare that they have no competing interests.

## Authors’ contributions

MD contributed to study design, carried out the National Survey of Children with Special Health Care Needs in Switzerland, performed statistical analyses, and drafted the manuscript. MMK designed and guided the National Survey of Children with Special Health Care Needs in Switzerland and helped to draft the manuscript. MAL contributed to study design, and helped to perform statistical analyses and draft the manuscript. All authors read and approved the final manuscript.
